# Preparation of gypsum with high purity and whiteness from phosphogypsum for CO_2_ mineral sequestration

**DOI:** 10.1038/s41598-023-28251-6

**Published:** 2023-03-13

**Authors:** Man Zhang, Xing Fan

**Affiliations:** School of Chemical Safety, North China Institute of Science and Technology, Langfang, 065201 China

**Keywords:** Carbon capture and storage, Environmental impact

## Abstract

Phosphogypsum (PG) is a solid waste product generated during wet-process phosphoric acid production. Various impurities considerably reduce the purity, whiteness, and application range of PG. This article analyzes the physical properties of PG in detail and systematically examines the content and distribution of impurities. Based on the obtained results, a simple process for the efficient removal of almost all impurities in the PG is proposed. The purity and whiteness of the purified gypsum (CaSO_4_) significantly increased to 99% and 92%, respectively. The migration of impurities and the material balance of this process were then analyzed. Most importantly, the purified gypsum showed high CO_2_ sequestration efficiency for CO_2_ mineral sequestration, through which a high value-added CaCO_3_ product was obtained.

## Introduction

Phosphogypsum (PG) is a hazardous industrial solid waste product from the wet phosphate industry^1,2^. The widespread deposition of that fine, light- to dark-grey, powder with a slight odor and strong acidity has been associated with serious environment-related issues. It is estimated that more than 200 million tons of PG are generated each year in the world^3^. PG is composed mainly of CaSO_4_·2H_2_O and impurities^4^. The presence of these impurities in PG significantly limits its application. The purity and the whiteness of PG are only approximately 85% and 50%, respectively. Improving the purity and whiteness of PG by removing such impurities would confer ideal inherent optical properties, and it can be used as a replacement for the ever-diminishing high-grade natural gypsum resource^5^.

The currently applied methods for impurity removal from phosphogypsum mainly eliminate the influence of phosphorus and fluorine impurities on gypsum-building materials^6–10^. However, phosphogypsum is produced predominantly in the suburbs, which limits its wider or high-end utilization and transportation distance^11^. In fact, purified phosphogypsum (PPG), which would be characterized by high purity and whiteness, would have good application prospects. Evidence exists that this purified gypsum (CaSO_4_) can be used as an additive or modifier in polymers, such as poly(vinyl chloride) (PVC) and polylactide^12^, as a high-quality chemical drying agent utilized for moisture removal^13^, or as a promising oxygen carrier for chemical-looping combustion (CLC)^14^.

In recent years, using PG as raw material for CO_2_ mineral sequestration has attracted significant research interest^15,16^. The calcium oxide content in PG is as high as 32%, which is a good raw material for capturing CO_2_. The product CaCO_3_ not only has a wide range of applications, but can also contribute to achieving permanent CO_2_ storage with a low detection risk. While the impurities in PG exert a great influence on the quality of the carbonated product and decrease carbonation conversion^17^, purified phosphogypsum is in high demand for both the production of value-added calcium carbonate and for CO_2_ mineral sequestration.

In this work, the mineralogical compositions, forms, and distribution of the impurities in PG were studied. Based on our results, we propose a simple and efficient impurity removal method that can be used to remove almost all impurities in PG. Further, the physical properties of purified gypsum were examined, the migration mechanism of impurities was analyzed, and the material balance of this process was established. Finally, the obtained purified gypsum was used to segregate CO_2_, a process by which calcium carbonate with high purity and whiteness was obtained. Furthermore, CO_2_ sequestration efficiency was also considerably improved.

## Experiments and analysis

### Materials and chemicals

The raw PG material used in this study was obtained from Sinochem Fuling Chemical Industry Co, Ltd. (Chongqing, China). After this, PG was dried at 40 °C for 12 h to remove the adsorbed water. It was placed in an airtight container and stored at room temperature until further analysis. The purified gypsum was then filtered, washed successively with deionized water, dried at 80 °C for 12 h, and placed in an airtight container. Analytical grade tributyl phosphate (TBP) and sulfuric acid were purchased from Sinopharm Chemical Reagent Co., Ltd.

### PG purification experiment

**Figure 1:**
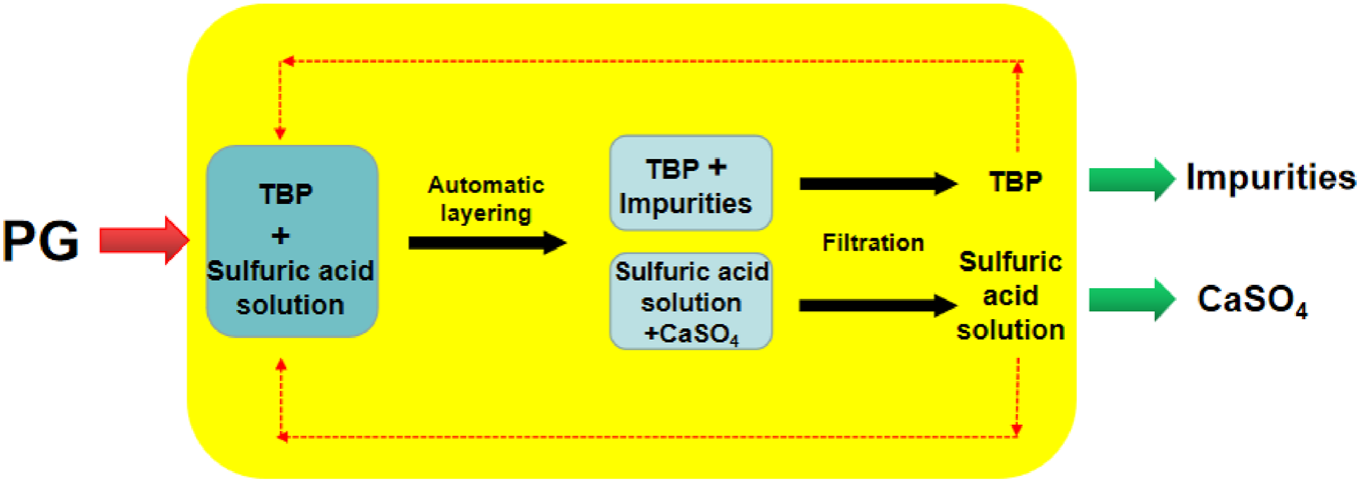
Schematic diagram of this PG purification experiment.

A PG purification experiment was performed under the following experimental conditions. We used H_2_SO_4_ with a concentration of 30% at a reaction temperature of 90 °C and a reaction time of 30 min. The sulfuric acid solution to PG weight ratio was 5:1, and the TBP to PG weight ratio was 5:1. The schematic diagram of this PG purification experimental process is presented in Fig. 1. First, a certain weight of PG was added to a closed stirred reaction kettle, in which sulfuric acid solution and tributyl phosphate (TBP) solvent were mixed. Further, the kettle was heated to the aforementioned reaction temperature at a certain stirring speed, and left to react for a certain period of time. The stirring was terminated after the reaction was completed. Because of the immiscibility, the aqueous sulfuric acid phase and the tributyl phosphate organic solvent phase were automatically separated, and the soluble PG impurities were dissolved. Next, acid-insoluble fine PG impurities were quickly encapsulated using tributyl phosphate, removed from the sulfuric acid solution, and transferred into the organic phase. The recrystallized anhydrous gypsum remained in the sulfuric acid solution. The acid-insoluble impurities were fine solids separated from the TBP solvent through filtration. The sulfuric acid slurry containing anhydrous gypsum in the lower layer was vacuum-filtered, washed with deionized water, and dried in an oven at 60 °C for 4 h to obtain high-quality PPG. The TBP solvent was recycled, and the sulfuric acid solution was also returned for continual PG decomposition. When it was recycled a certain number of times, it was returned to the dihydrate wet-process phosphoric acid system for phosphate rock decomposition. This process hardly causes any secondary pollution to the environment. The principle of interaction between TBP and acid-insoluble fine impurities, and the optimization of process conditions are not described in this paper.

### Purified gypsum mineral carbonation experiment

After removing impurities, the purified gypsum could be used to capture CO_2_ and prepare a high-value-added CaCO_3_ product. Enhancing carbonation with ammonia under increased CO_2_ pressure was employed to shorten the reaction time. The process can be expressed as follows:

1 $${\text{CaSO}}_{{4}} \left( {\text{s}} \right) \, + {\text{ CO}}_{{2}} \left( {\text{g}} \right) \, + {\text{ 2NH}}_{{3}} \left( {\text{g}} \right) \, + {\text{ H}}_{{2}} {\text{O }}\left( {\text{l}} \right) \to {\text{CaCO}}_{{3}} \left( {\text{s}} \right) \, + \, ({\text{NH}}_{{4}} )_{{2}} {\text{SO}}_{{4}} \left( {{\text{aq}}} \right).$$

The aqueous mineral carbonation reaction was carried out inside a 2-L high-pressure stirred tank. This tank was connected to a CO_2_ gas inlet line which provided CO_2_ gas (99.9%) from a gas cylinder. The stirred tank was equipped with a pressure transducer which was connected to a data acquisition system and displayed the real-time inside pressure of the stirred tank. The temperature was regulated by a heating jacket and was recorded by a thermocouple. The specific conditions under which the reaction occurred were as follows: CO_2_ partial pressure 0.8 MPa, initial temperature 80 °C, stirring speed 300 rpm, excess ammonia ratio 1.2, and liquid–solid ratio 2:1. The detailed description of the experimental conditions was previously reported^4^. The purity of CaCO_3_ (*P*_CaCO3_ (wt%)) in the solid product was calculated based on the content of carbon in CaCO_3_. Here, Mw denotes molecular weight. The carbonation conversion ratio (*X*_rate_) was calculated by the value of the purity of CaCO_3_ determined in the solid product. The higher purity of the calcium carbonate increased the conversion ratio.

2 $$P_{{{\text{CaCO}}_{{3}} }} \left( {{\text{wt\% }}} \right) = \frac{{ \, P_{{\text{C}}} {\text{(wt\% )}} \times {\text{Mw}}_{{^{{{\text{CaCO}}_{{_{{3}} }} }} }} }}{{{\text{Mw}}_{{\text{C}}} }},$$

3 $$X_{{{\text{rate}}}} \left( {\text{\% }} \right) = \frac{{{\text{ CaCO}}_{{3}} {\text{ purity in solid product}}}}{{{\text{estimated CaCO}}_{{3}} {\text{ purity in 100\% carbonated solid product}}}} \times 100.$$

### Characterization analysis

The surface morphology of the raw phosphogypsum (PG) and the PPG was examined by scanning electron microscopy (JSM-6700F, JEOL Japan). The chemical composition and crystal type of PG and PPG were determined by X-ray fluorescence (XRF, AXIOS, PANalytical BV, Almelo, The Netherlands) and X-ray diffraction (XRD, X'Pert Pro, PANalytical B.V. Netherlands) analyses. The internal structure of the raw PG and the internal element distribution of S, Si, P, Fe, Ba, and Ti were examined with a Mineral Liberation Analyzer (MLA-250, FEI Company, Hillsboro, OR, USA). The sample was embedded in epoxy resin mounts, polished, and covered with a carbon layer prior to analysis. The whiteness was tested according to the GB/T 5950-2008 standard. The average particle size of the CaCO_3_ produced by PPG mineral carbonation was determined with a Malvern laser particle size analyzer (Mastersizer 2000, Malvern, UK).

## Results and discussion

### The morphology, crystal form, composition, and whiteness of the PG raw material

**Figure 2:**
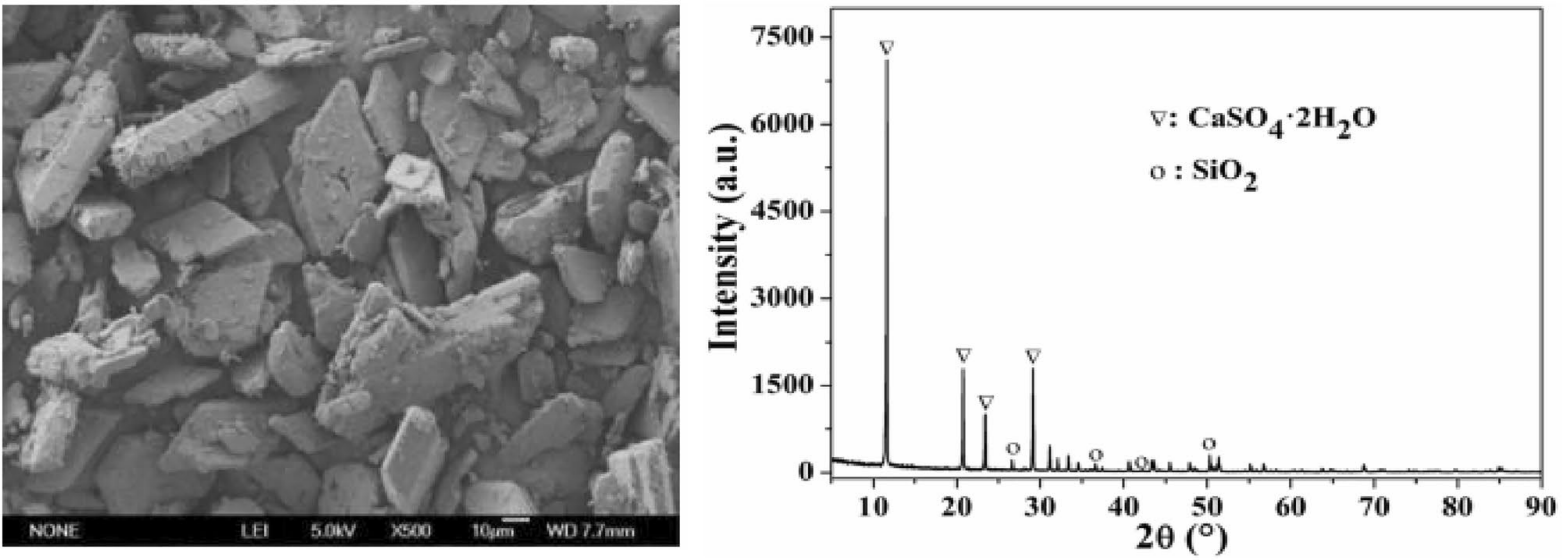
SEM photograph and XRD pattern of PG.

An SEM photomicrograph showing the surface morphology of PG is given in Fig. 2. As can be seen, the surface of the parallelogram or rhombic crystal is rough, indicating that many small impurities have adhered to the PG surface. The main crystal phase of PG is CaSO_4_·2H_2_O. The characteristic peaks of quartz, which is the main impurity in PG, were also detected. The XRD pattern could not detect other impurities due to their low content. The chemical composition of PG determined by XRF is presented in Table 1. SO_3_ (56.92%) and CaO (33.64%) were determined as major constituents. A high CaO content indicates a high potential for mineral carbonation for CO_2_ sequestration. SiO_2_ had the highest percentage of all impurities, accounting for 6.3%. Phosphorus (0.71%) and fluorine (0.91%) impurities were also detected in our study; they have seriously affected phosphogypsum application worldwide. As visible in Table 1, due to the influence of various impurities, the whiteness of PG was only 45%.

**Table 1:** Chemical composition and whiteness of PG.

	CaO	SO_3_	SiO_2_	Al_2_O_3_	P_2_O_5_	Fe_2_O_3_	F	Na_2_O	MgO	K_2_O	TiO_2_	SrO	BaO	Whiteness
wt%	33.64	56.92	6.30	0.64	0.71	0.33	0.91	0.06	0.09	0.15	0.11	0.06	0.06	45%

### The distribution of the impurities in PG

**Figure 3:**
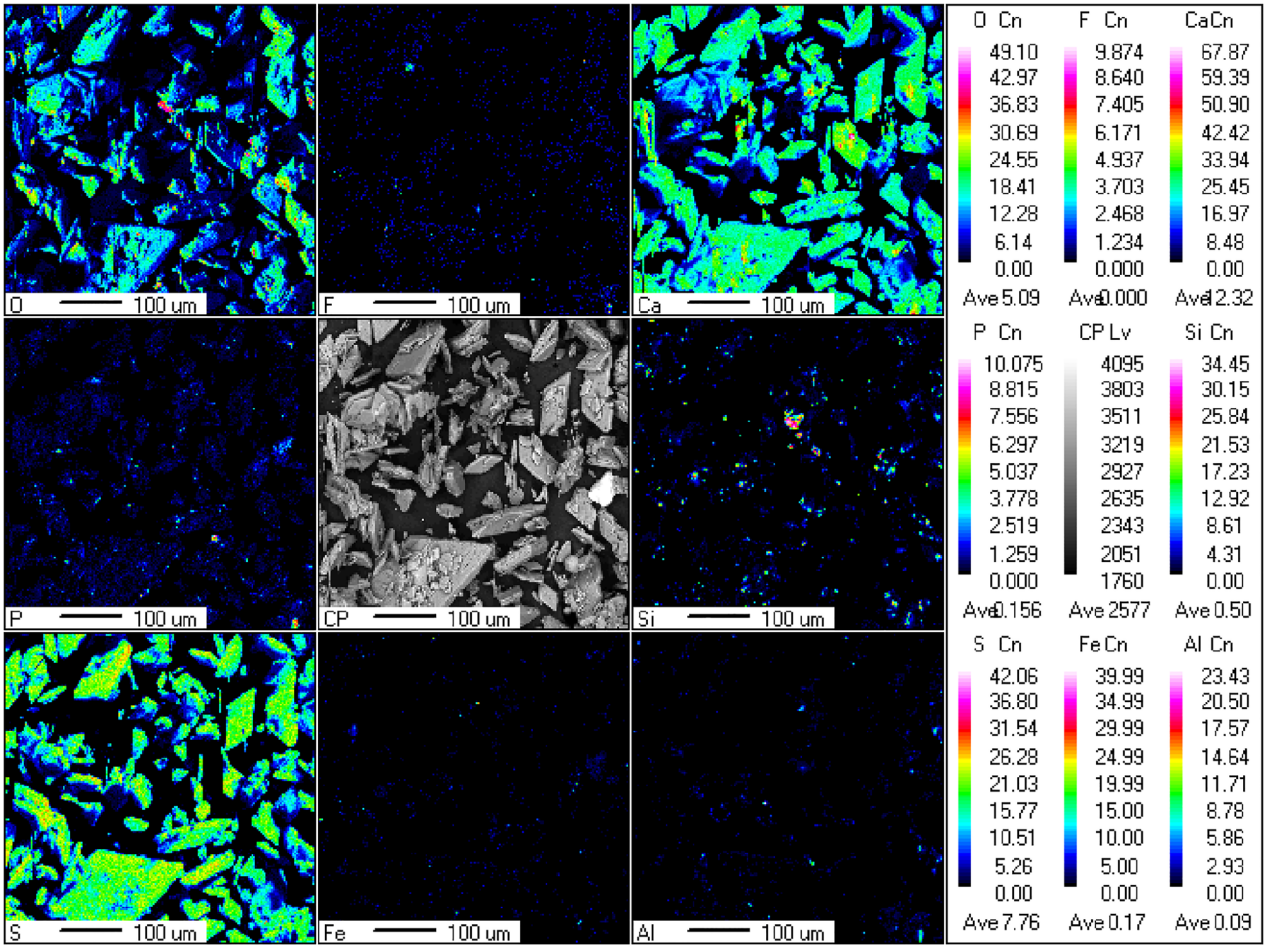
The distribution of Ca, S, O, F, P, Si, Al, and Fe on the surface of PG, as determined by EPMA.

**Figure 4:**
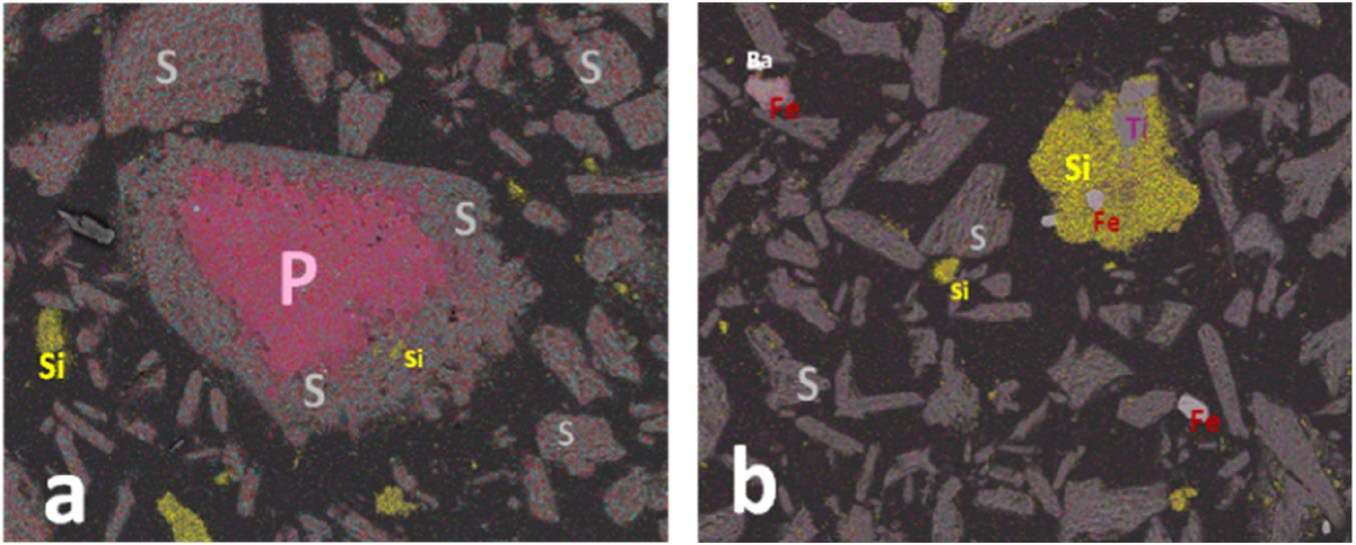
The distribution of the impurities P and Si wrapped in PG (**a**) and the impurities Fe and Ti wrapped in quartz phase (**b**), as determined by EDS.

Figure 3 displays the distribution of the main elements (Ca, S, O, F, P, Si, Al, and Fe) on the surface of PG determined by EPMA. It can be seen that the distribution of Ca, S, and O is consistent with PG morphology, further indicating the existence of a crystal phase of PG, which is composed of CaSO_4_·2H_2_O. However, the distribution of F, P, Si, Fe, and Al was uneven, reflecting abundant impurities mixed with PG or attached to its surface. Figure 4 illustrates the internal structure of raw PG and the internal element distribution of S, Si, P, Fe, Ba, and Ti detected by SEM–EDS. As shown in Fig. 4a, some impurities (P and Si) were also wrapped in PG. Besides, other impurities (Ti and Fe) were wrapped in the quartz phase, as visible in Fig. 4b. Therefore, we concluded that these impurities are dispersed in PG, and their contents vary significantly. Furthermore, impurities such as unreacted phosphate rock and quartz may be encapsulated in PG, which hinders the efficient removal of PG impurities.

### Analysis of purified gypsum

**Figure 5:**
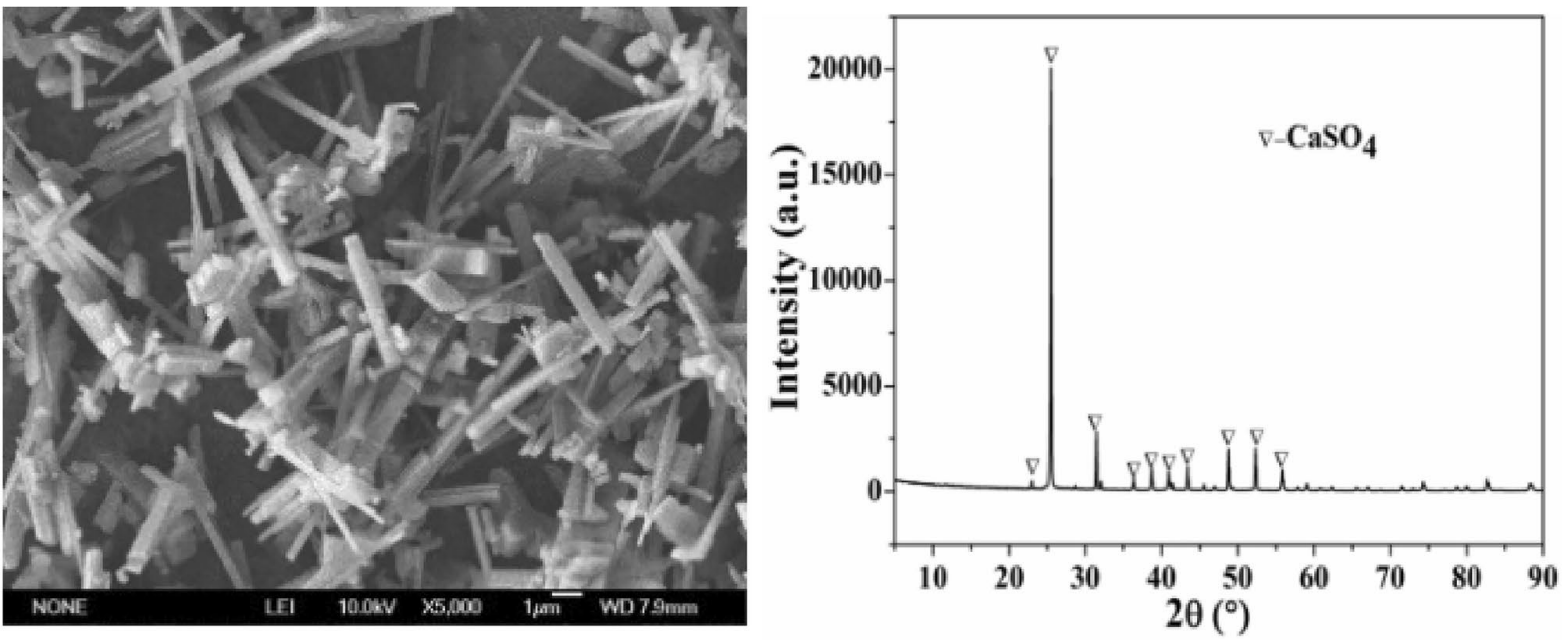
A SEM photograph and the XRD pattern of PPG.

Based on the characteristics of the above-mentioned impurities and the concept for the development of a simple and efficient removal of PG impurities, in this study, we adopted the method of dissolving and removing soluble impurities in sulfuric acid solution. Then, TBP was used to remove insoluble impurities to obtain high-quality gypsum. An SEM photomicrograph and the XRD pattern of PPG are displayed in Fig. 5. As can be seen from the figure, the surface of the clubbed crystal in PPG has no small impurities attached to the surface and is smoother than that in PG. The sharp characteristic peaks of CaSO_4_ were detected. The crystal phase of PPG consisted of pure CaSO_4_, without other characteristic peaks of impurities, revealing that PG had undergone crystal transformation from CaSO_4_·2H_2_O to CaSO_4_ through dissolution and recrystallization in sulfuric acid solution. These chemical processes promoted the dissolution of CaSO_4_·2H_2_O and the subsequent release of higher quantities of Ca^2+^ and SO_4_^2−^. Then, Ca^2+^ reacted and quickly bound to SO_4_^2−^ to form CaSO_4_ crystals and precipitate (Reaction 2). Hence, CaSO_4_ with high purity and whiteness was obtained by the elimination of impurities. The process can be described by the following equation:

4 $${\text{CaSO}}_{{4}} \cdot{\text{2H}}_{{2}} {\text{O}} \to {\text{Ca}}^{{{2} + }} + {\text{ SO}}_{{4}}^{{{2} - }} + {\text{ 2H}}_{{2}} {\text{O}} \to {\text{CaSO}}_{{4}} + {\text{ 2H}}_{{2}} {\text{O}}.$$

The chemical composition of PPG is presented in Table 2. It can be seen that the contents of the main impurities in PPG, including SiO_2_, P_2_O_5_, Al_2_O_3_, and Fe_2_O_3_, were 0.12%, 0.01%, 0.02%, and 0.02%, respectively. The content of F was below the detection limits. Therefore, it can be concluded that almost all the impurities were effectively removed. Moreover, because of the radical decrease in the contents of impurities, the whiteness of PPG remarkably increased from 45 to 92.71%.

**Table 2:** Chemical composition and whiteness of PPG.

	CaO	SO_3_	SiO_2_	Al_2_O_3_	P_2_O_5_	Fe_2_O_3_	F	Na_2_O	MgO	K_2_O	TiO_2_	SrO	BaO	Whiteness
PPG	35.94	63.71	0.12	0.02	0.01	0.02	n.d.	n.d.	n.d.	n.d.	0.05	0.05	0.04	92%

*n.d.* not detected.

Data on the amounts of PG, the produced PPG with impurities, and the contents of the latter leached into the sulfuric acid solution are listed in Table 3. The results of material balance show that approximately 59.17 kg of impurities and 661.93 kg of PPG were respectively separated per ton of PG. Approximately 278.78 kg of other matters were solved into the sulfuric acid solution, including 180.1 kg water, 41.43 kg SO_3_, 37.53 kg CaO, 9.6 kg P_2_O_5_, 5.95 kg SiO_2_, 1.4 kg Al_2_O_3_, 0.73 kg Fe_2_O_3_, and a very small amount of F, Na_2_O, K_2_O, MgO, and TiO_2_. Interestingly, almost all of the P_2_O_5_ was leached into the sulfuric acid solution, whereas most of F and SiO_2_ were extracted into the TBP organic phase, indicating that the impurities of P_2_O_5_, F, and SiO_2_ in PG can be further economically recovered.

**Table 3:** Material balance established in this impurity removal experiment.

Contents, kg	PG	PPG	Impurities	Sulfuric acid solution
CaO	275.74	237.99	0.22	37.53
SO_3_	466.56	421.89	3.24	41.43
SiO_2_	49.18	0.79	42.44	5.95
Al_2_O_3_	5.24	0.13	3.71	1.4
P_2_O_5_	9.84	0.07	0.17	9.6
Fe_2_O_3_	2.7	0.13	1.84	0.73
F	5.82	0	5.32	0.5
Na_2_O	0.49	0	0.04	0.45
MgO	0.74	0	0.41	0.33
K_2_O	1.23	0	0.97	0.26
TiO_2_	0.9	0.33	0.27	0.3
Others	1.34	0.6	0.1	0.2
H_2_O	180.1	0	0.54	180.1
Total	999.88	661.93	59.17	278.78

### Purified gypsum mineral carbonation for CO_2_ sequestration

**Figure 6:**
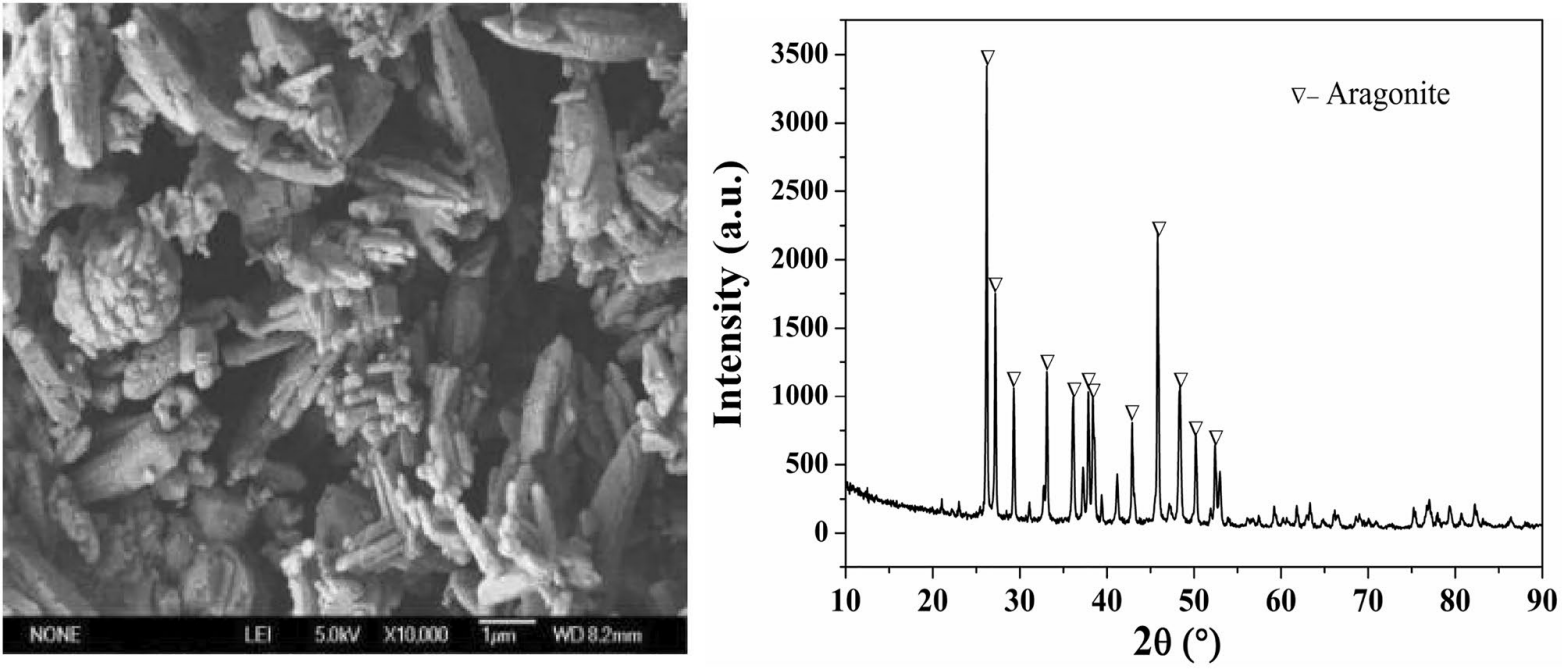
A SEM photograph and the XRD pattern of the CaCO_3_ product obtained from PPG through mineral carbonation.

The process of mineral carbonation of PPG was investigated to achieve effective utilization of this calcium sulphate with high purity and whiteness. The approach described here has the potential to solve simultaneously two major environmental problems (the pollution problem of PG stacking and the greenhouse effect caused by CO_2_ emissions) while manufacturing high value-added chemical products with low energy expenditure and costs. An SEM photomicrograph and the XRD pattern of the CaCO_3_ product from PPG through mineral carbonation are provided in Fig. 6. As can be seen from the figure, the surface of the CaCO_3_ product obtained from PPG has no impurities attached to its surface and is smoother than the CaCO_3_ product obtained from PG. Notably, the crystal phase of the CaCO_3_ product obtained from PPG was found to have been converted from calcite to aragonite. Lu et al.^18^ found that when impurities, such as F^−^, Fe^3+^, and Mg^2+^ (especially F^−^), were present in phosphogypsum, the carbonation reaction was more likely to form thermodynamically stable calcite. Since impurities in the phosphogypsum, such as F^-^, had been previously removed by high-efficiency purification, the influence of impurities on the carbonation reaction was eliminated. Thus, the crystal form of the product calcium carbonate was transformed into aragonite calcium carbonate.

As visible from Table 4, the purity and whiteness of the produced CaCO_3_ from PPG reached 99.1% and 91.8%, respectively, whereas the purity and whiteness of the CaCO_3_ obtained from PG were only 86.5% and 47.8%, correspondingly. These results indicated that after removing the PG impurities by this purification technology, the quality of the CaCO_3_ product was greatly improved. Besides, the average particle size of the produced CaCO_3_ decreased from 17 to 6.2 μm, suggesting that this CaCO_3_ can be widely used as a valuable filler. Moreover, the carbonation conversion increased only slightly from 97 to 99.5%, whereas a considerable rise was achieved in the sequestrated CO_2_ amount, from 224 to 322 kg per ton of PG and PPG, respectively. Therefore, the PG purification using the suggested method here substantially improved the CO_2_ sequestration efficiency. Furthermore, PG mineral carbonation for CO_2_ sequestration is of great economic feasibility due to the generation of a high-value-added CaCO_3_ product and its high CO_2_ sequestration efficiency.

**Table 4:** Comparison between the CaCO_3_ product obtained from PG and the CaCO_3_ product generated from PPG through mineral carbonation.

Index		PG	PPG
CaCO_3_ product	Purity, %	86.5	99.1
	Whiteness, %	47.86	91.89
	Average particle size, μm	17.0	6.2
Carbonation conversion, %		97	99.5
Sequestrated CO_2_, kg/t		224	322

## Conclusions

In this work, we analyzed the physical properties of PG and systematically studied the content and distribution of its impurities. The results showed that SiO_2_ was the largest contributor (6.3%) to the significant amounts and variety of impurities in PG, followed in descending order by fluorine (0.91%) and phosphorus (0.71%). The greatly varying content of dispersed impurities has seriously limited PG practical applications, reducing its whiteness to only 45%. Moreover, the unreacted phosphate rock and quartz impurities may be encapsulated in PG, which hinders the impurity removal efficiency. The impurity removal method proposed here leads to the dissolution of the soluble PG impurities. Notably, acid-insoluble fine impurities in PG are quickly encapsulated by tributyl phosphate and removed from the sulfuric acid solution into the organic phase. As a result, the impurity content in purified gypsum is greatly reduced, significantly increasing its purity to more than 99% and its whiteness to more than 92%. Therefore, CaSO_4_ with high purity and high whiteness can be obtained. It is estimated that approximately 59.17 kg of impurities and 661.93 kg of CaSO_4_ can be separated per ton of PG. PPG is further used to sequestrate CO_2_, resulting in the production of a high-value-added CaCO_3_ product and achieving high CO_2_ sequestration efficiency. Therefore, the economic feasibility of PG mineral carbonation for CO_2_ sequestration is significantly promoted.

## Data Availability

The datasets used and/or analysed during the current study available from the corresponding author on reasonable request.

## References

[1] Silva LFO (2022). A review on the environmental impact of phosphogypsum and potential health impacts through the release of nanoparticles. Chemosphere.

[2] Cui Y, Chang IS, Yang S, Yu X, Cao Y, Wu J (2022). A novel dynamic business model to quantify the effects of policy intervention on solid waste recycling industry: A case study on phosphogypsum recycling in Yichang, China. J. Clean. Prod..

[3] Pu S, Zhu Z, Huo W (2021). Evaluation of engineering properties and environmental effect of recycled gypsum stabilized soil in geotechnical engineering: A comprehensive review. Resour. Conserv. Recy..

[4] Zhao H, Li H, Bao W, Wang C, Li S, Lin W (2015). Experimental study of enhanced phosphogypsum carbonation with ammonia under increased CO_2_ pressure. J. CO2 Util..

[5] Jia R, Wang Q, Luo T (2021). Reuse of phosphogypsum as hemihydrate gypsum: The negative effect and content control of H_3_PO_4_. Resour. Conserv. Recy..

[6] Wang J (2020). A novel method for purification of phosphogypsum. Physicochem. Probl. Min..

[7] Cai Q (2021). Efficient removal of phosphate impurities in waste phosphogypsum for the production of cement. Sci. Total Environ..

[8] Cao W, Yi W, Li J, Peng J, Yin S (2021). A facile approach for large-scale recovery of phosphogypsum: An insight from its performance. Constr. Build. Mater..

[9] Fan P (2022). The Influences of soluble phosphorus on hydration process and mechanical properties of hemihydrate gypsum under deep retarding condition. Materials.

[10] Du M (2022). The study on the effect of flotation purification on the performance of α-hemihydrate gypsum prepared from phosphogypsum. Sci. Rep. U.K..

[11] Chowdhury A, Naz A (2021). Waste to resource: Applicability of fly ash as landfill geoliner to control ground water pollution. Mater. Today Proc..

[12] Kondratieva N, Barre M, Goutenoire F, Sanytsky M, Rousseau A (2020). Effect of additives SiC on the hydration and the crystallization processes of gypsum. Constr. Build. Mater..

[13] Misnikov O (2018). The hydrophobic modification of gypsum binder by peat products: Physico-chemical and technological basis. Mires Peat.

[14] Abuelgasim S, Wang W, Abdalazeez A (2021). A brief review for chemical looping combustion as a promising CO_2_ capture technology: Fundamentals and progress. Sci. Total Environ..

[15] Alfonso D (2022). High-performance ligand-protected metal nanocluster catalysts for CO_2_ conversion through the exposure of undercoordinated sites. Catalysts.

[16] Calderón-Morales BR, García-Martínez A, Pineda P, García-Tenório R (2021). Valorization of phosphogypsum in cement-based materials: limits and potential in eco-efficient construction. J. Build. Eng..

[17] Liu W (2021). CO_2_ mineral carbonation using industrial solid wastes: A review of recent developments. Chem. Eng. J..

[18] Lu H, Zhong B, Liang B, Zhang Y (2002). Effect of fluorine, iron and magnesium impurities on the conversion process of phosphogypsum mineral carbonation. J. Chem. Eng. Chin. Univ..

